# Supplementary material for: Simple Rules for Complex Near-Glass-Transition Phenomena in Medium-Sized Schiff Bases
Source: Int J Mol Sci. 2022 May 6;23(9):5185. doi: 10.3390/ijms23095185 (PMC9103181; doi:10.3390/ijms23095185)
Supplement: Supplementary file 1 [file ijms-23-05185-s001.zip › ijms-1698741-supplementary.pdf]

Simple Rules For Complex Near-Glass-Transition Phenomena in Medium-Sized Schiff Bases

Supplementary Materials

Andrzej Nowok [1], Wioleta Cieślik [2], Joanna Grelska [3,4], Karolina Jurkiewicz [3,4], Natalina Makieieva [5], Teobald Kupka [5], José Alemán [6,7], Robert Musioł [2,*], and Sebastian Pawlus [3,4]

1. Department of Experimental Physics, Wrocław University of Science and Technology, 50-370 Wrocław, Poland
2. Institute of Chemistry, University of Silesia in Katowice, 75 Pułku Piechoty 1A, 41-500 Chorzów, Poland
3. August Chełkowski Institute of Physics, University of Silesia in Katowice, 75 Pułku Piechoty 1, 41-500 Chorzow, Poland
4. Silesian Center for Education and Interdisciplinary Research, 75 Pułku Piechoty 1A, 41-500 Chorzow, Poland
5. Opole University, Faculty of Chemistry, Oleska Street 48, 45-052 Opole, Poland
6. Department of Organic Chemistry, Universidad Autónoma de Madrid, Calle Francisco Tomás y Valiente, 7, Cantoblanco, 28049 Madrid, Spain
7. Institute for Advanced Research in Chemical Sciences (IAdChem), Universidad Autónoma de Madrid, 28049 Madrid, Spain

*robert.musiol@us.edu.pl

Table of contents

1. Additional information for the molar mass – glass transition temperature relationship

2. The Avramov model

3. Fragility index of the previously published glycine imino esters

1. Additional information for the molar mass – glass transition temperature relationship

Table S1. Molar mass (*M*) and glass transition temperatures (T_g) for glycine imino esters and selected previously published compounds with C=N bonds.

Compound	*M* (g/mol)	T_g (K)	Ref.
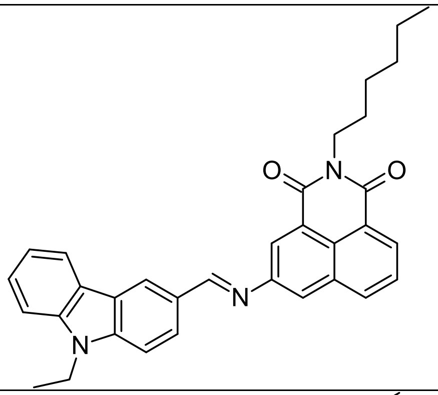	502	328	34
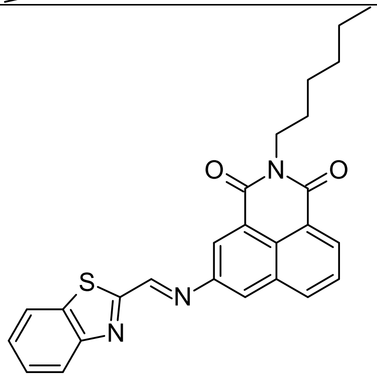	442	308	34
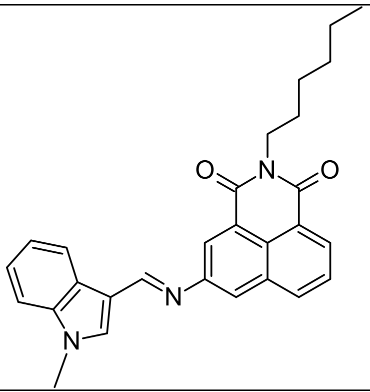	437.5	320	34

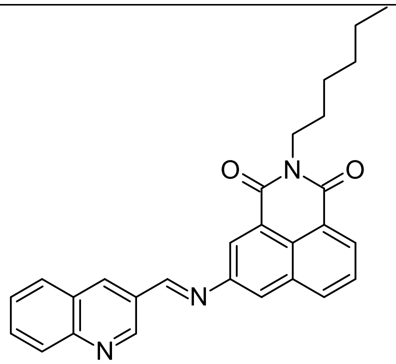	436	301	34
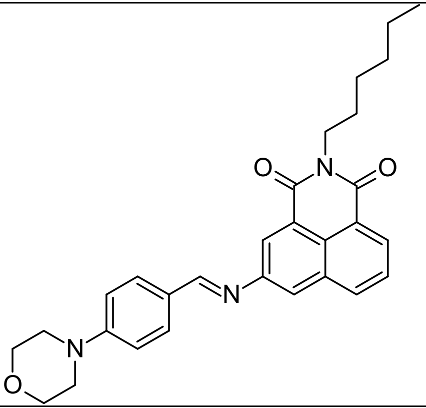	470	314	34
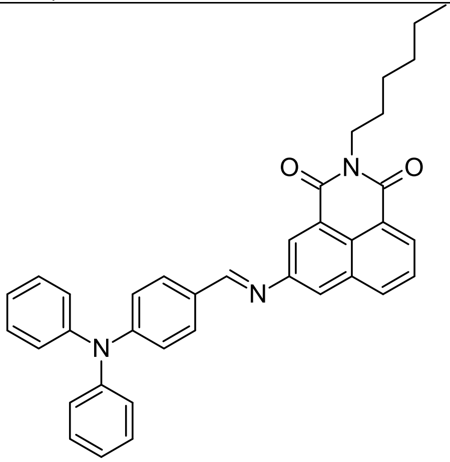	552	324	34
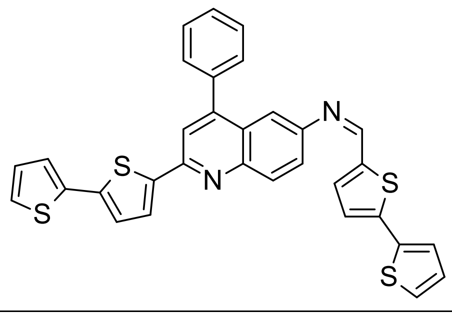	561	361	35

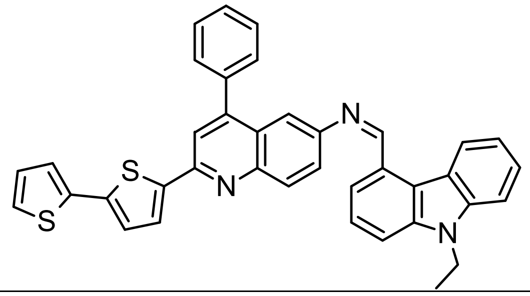	590	463	35
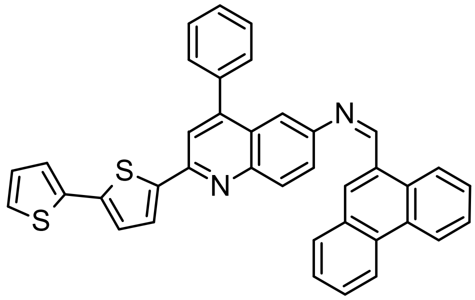	573	445	35
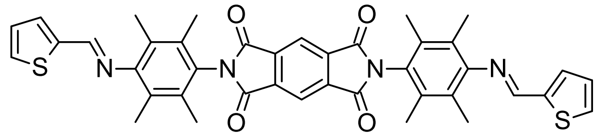	699	490	8
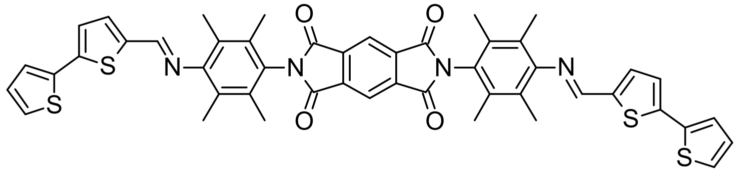	863	449	8
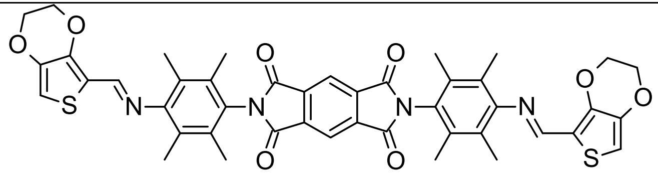	815	514	8
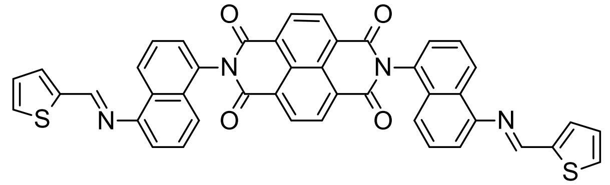	737	550	8
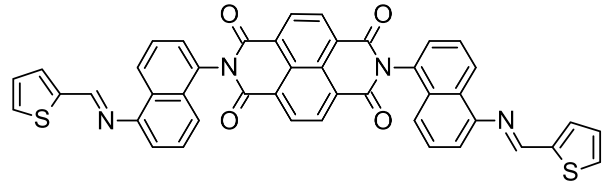	901	564	8
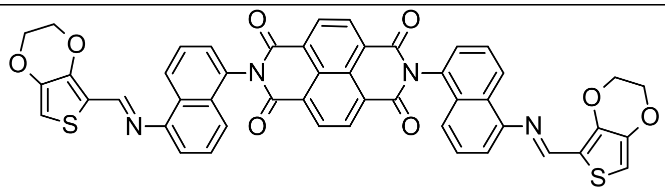	853	551	8

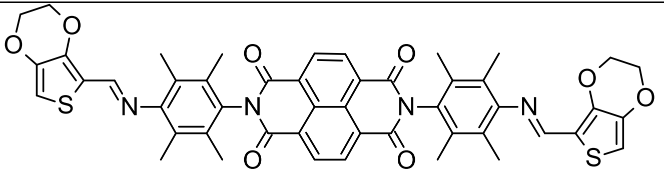	865	464	8
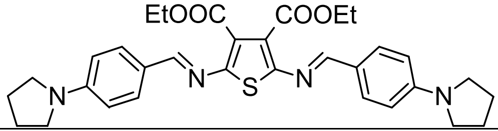	573	352	6
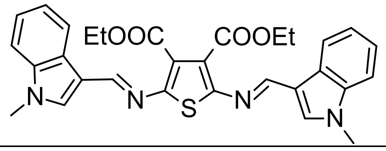	541	363	6
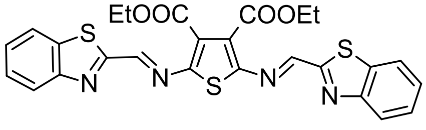	549	372	6
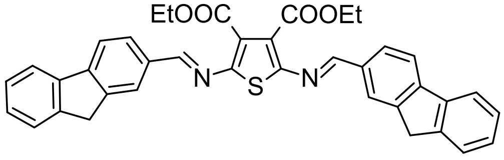	611	363	6
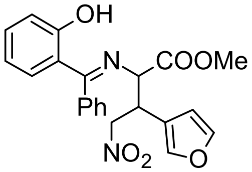	408	285	21
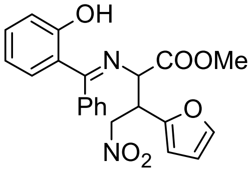	408	285	21
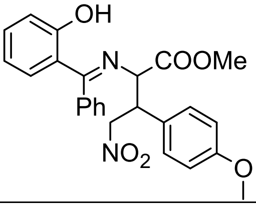	448	301	21
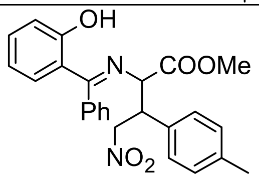	432	304	21
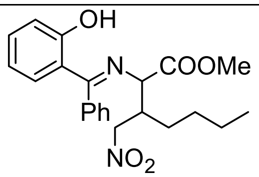	398	264	-

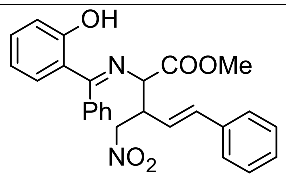	444	304	-

2. The Avramov model

In order to fit the pressure-temperature surface of relaxation times for compounds **1** and **2**, the Avramov model was applied. This model was originally formulated for pressure-temperature evolution of viscosity, but was adapted to deal with nonlinear, super-Arrhenius pressure and temperature dependences of dielectric relaxation times [52]. According to this model, the p-T plane of τ_{Max} can be parametrized by the following equation of state:

$$log\tau_{Max} = log\tau_{\infty} + \left(\frac{T_{ref}(p_0)}{T}\right)^{F}\left(1+\frac{p}{\Pi}\right)^{\beta} log\left(\frac{\tau_{ref}}{\tau_{\infty}}\right) \tag{1}$$

In this equation τ_{∞} is the minimum limit value of the relaxation time achieved at $T \to \infty$ and $p \to 0$, p_0 is the atmospheric or zero pressure, $T_{ref}(p_0)$ is a reference temperature at p_0, Π is the internal pressure of the system, τ_{ref} is the relaxation time at T_{ref}, whereas F, and β are thermodynamic constants given by the expressions:

$$F = F_0\left(1-\frac{C}{C_{p_0}} ln\left(\frac{\Pi+p}{\Pi+p_0}\right)\right) \approx F_0\left(1-\frac{C}{C_{p_0}} ln\left(1+\frac{p}{\Pi}\right)\right) \tag{2}$$

$$F_0 = \frac{2C_{p_0}}{ZR} \tag{3}$$

$$\beta = \frac{2\kappa_0 V_m}{ZR}\Pi \tag{4}$$

Herein, F is the so-called fragility parameter, C is a constant, C_{P0} is the heat capacity at p_0, R is the gas constant, Z is the number of channels along which the building units can leave a given position, κ_0 is the thermal expansion coefficient and V_m is the molar volume [52].

During fitting procedure, $\log\tau_\infty$, F, Π and β parameters were used as free variables, whereas $T_{ref}(p_0)$ was glass transition temperature under ambient pressure. Finally, $\log\tau_{ref}$ was taken as $\log\tau_{ref} = 2$ because the structural relaxation time at T_g is equal 100 s for low-weight glass-formers.

3. Fragility index of the previously published glycine imino esters

Ambient-pressure fragility index, m_p, was calculated for the previously published glycine imino esters based on their temperature dependences of structural relaxation times that can be found in Ref. 21. The obtained values are presented in Table S2.

Table S2. Calculated ambient-pressure fragility index, m_p, for the previously published glycine imino esters.

Compound	m_p
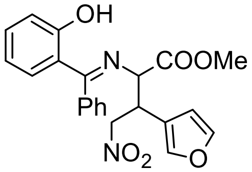	84 ± 2
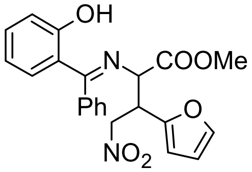	84 ± 2
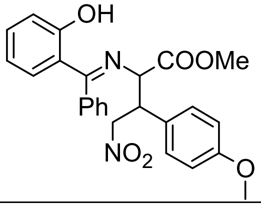	81 ± 2
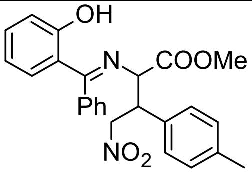	80 ± 2
